# Standardising Pleural Management in Suspected Malignant Pleural Effusion Using a Structured Pre-Test Probability and RED Score-Guided Proforma: A Single-Centre Before-and-After Quality Improvement Project

**DOI:** 10.7759/cureus.116793

**Published:** 2026-09-23

**Authors:** Ahmed R Fadel, Hashim Al-Sarireh, Alma Alrayyes, Narendra Chinnappa

**Affiliations:** 1 Respiratory Medicine, Swansea Bay University Health Board, Swansea, GBR; 2 General Medicine, Swansea Bay University Health Board, Swansea, GBR

**Keywords:** indwelling pleural catheter, malignant pleural effusion, medical thoracoscopy, pleural services, quality improvement projects, red score

## Abstract

Background

The management of suspected malignant pleural effusion (MPE) is often variable, with many patients undergoing repeated pleural aspirations before a definitive management decision is made. The RED (respiratory rate, effusion depth and dyspnoea) score is a recently derived and validated tool that predicts the time to the next pleural fluid drainage procedure. We describe a single-center, before-and-after quality improvement project (QIP) evaluating the introduction of a structured proforma incorporating clinical pre-test probability and the RED score into routine pleural clinic assessment.

Methods

A retrospective baseline cycle (Cycle 1, n=48) was compared with a prospective intervention cycle (Cycle 2, n=26) following the implementation of a structured assessment pro forma. The primary outcome was the proportion of patients experiencing unplanned emergency admission or earlier-than-planned pleural clinic review within three months of the index pleural procedure. Secondary outcomes included the proportion of patients requiring more than two pleural procedures and the proportion of patients achieving definitive pleural management within three months. Categorical variables were compared using Fisher's exact test given the small sample sizes; results are presented with risk differences, 95% confidence intervals, and odds ratios where calculable. No a priori power calculation was performed, and this exploratory before-and-after design does not permit causal inferences.

Results

In Cycle 1, 16/48 patients (33.3%) experienced an emergency admission or unplanned pleural clinic review, compared with 2/26 (7.7%) in Cycle 2 (Fisher's exact test, P=0.021; risk difference −25.6%, 95% CI −42.5 to −8.8; odds ratio 6.0). The proportion of patients requiring more than two pleural procedures fell from 33.3% to 22.7% (P=0.415, not statistically significant). Cytology was positive in 24/48 (50.0%) Cycle 1 patients and 10/26 (38.5%) Cycle 2 patients. The two cohorts differed descriptively in performance status (Eastern Cooperative Oncology Group (ECOG) ≥2:41.7% vs. 87.5%, P<0.001) and malignancy type distribution. RED scores were documented only anecdotally within free-text clinical notes for a subset of Cycle 2 patients and were not systematically recorded or analyzed as a distribution.

Conclusions

A structured pleural assessment pathway was associated with a statistically significant reduction in emergency presentations and a non-significant reduction in repeated procedures. However, given the small, single-center, non-randomised, uncontrolled before-and-after design, differing case mix between cohorts, and incomplete RED score documentation, these findings should be regarded as preliminary and hypothesis-generating. They support further, larger, multicenter evaluations rather than immediate wider adoption.

## Introduction

Malignant pleural effusion (MPE) is one of the most common and distressing complications of advanced malignancies, contributing substantially to breathlessness, reduced quality of life, and unplanned hospital utilisation [1-3]. MPE most often arises from metastatic spread to the pleura, typically from lung or breast cancer, and its presence is associated with advanced-stage disease and poor survival; in lung cancer, malignant effusion upstages the disease to Stage 4 [1]. Diagnosis requires the exclusion of infection and confirmation via imaging and, where possible, pleural fluid cytology or biopsy, with thoracic ultrasound offering additional value in identifying diaphragmatic or pleural thickening and nodularity suggestive of malignancy [1].

Despite established guidance recommending early definitive management (e.g., indwelling pleural catheter (IPC) insertion, pleurodesis, or medical thoracoscopy) in confirmed or highly suspected MPE, management in routine practice remains highly variable, and many patients undergo repeated diagnostic or therapeutic aspirations before a definitive strategy is pursued [4-6]. This delay is a recognised driver of avoidable hospital presentations, and several authors have cautioned against delaying IPC placement once malignancy is confirmed or highly suspected [7]. A systematic review of IPC efficacy and safety relative to the timing of cancer therapy further reinforces that earlier catheter placement, appropriately sequenced with oncological treatment, improves symptom control without increasing the complication rates [8].

Contemporary diagnostic approaches increasingly emphasise the combination of clinical pre-test probability with objective prognostic tools rather than relying on cytology alone, as cytology can be falsely negative in a substantial proportion of true MPE cases [2,9]. Thoracic ultrasound has become a core adjunct to this assessment, allowing bedside characterization of effusion size, septations, and pleural nodularity, which informs both diagnostic likelihood and procedural planning [10]. The RED (respiratory rate, effusion depth and dyspnoea) score is a recently derived and externally validated bedside tool that predicts the time to the next ipsilateral pleural fluid drainage procedure in patients undergoing therapeutic aspiration for known or suspected MPE. It is calculated from three readily available variables at the time of aspiration: respiratory rate, effusion depth measured on thoracic ultrasound, and dyspnea severity measured on a visual analog scale, with a supplementary point added for a respiratory rate of ≥22 breaths/min. Patients are then stratified into low, medium, or high RED score categories, corresponding to an increasing likelihood of requiring a further pleural procedure within six weeks, two to six weeks, or two weeks, respectively [11]. This reflects a broader shift toward personalised prognostication in MPE, as a uniform, one-size-fits-all approach to the timing of intervention is increasingly recognised as inadequate, given the heterogeneity of underlying malignancies and patient trajectories [12,13]. Multidisciplinary input from respiratory, oncology, and palliative care teams further supports individualised decision-making, particularly in complex cases such as MPE secondary to malignant pleural mesothelioma [14,15].

Breathlessness remains the dominant symptom driving both diagnosis and re-presentation in MPE, and its trajectory should inform the intensity and speed of the intervention chosen for each patient [16]. Rarely, MPE may spontaneously improve without intervention, as documented in isolated case reports, underscoring the heterogeneity of disease behavior and the importance of individualised, rather than protocolised, timelines for intervention [17].

## Materials and methods

Study design

This was a single-centre, before-and-after quality improvement project (QIP), not a randomised or controlled study, conducted in a UK district general hospital across two sequential, non-overlapping six-month cycles: a retrospective baseline cycle (Cycle 1) and a prospective intervention cycle (Cycle 2). We explicitly identified this as an uncontrolled before-and-after design; no concurrent control group was used, and no randomization or blinding of clinicians or outcome assessors to cycle allocation was possible or attempted.

Quality improvement project (QIP) methodology (PDSA framework)

This project followed an informal Plan-Do-Study-Act (PDSA) improvement structure.

*Plan*: The baseline practice was characterised retrospectively (Cycle 1) to quantify unplanned emergency presentations and repeated procedures.

*Do*: A structured proforma incorporating pre-test probability, thoracic ultrasound findings, dyspnea visual analog scale, Eastern Cooperative Oncology Group (ECOG) performance status, and RED score was introduced into the routine pleural clinic workflow.

*Study*: Outcomes in the prospective cohort (Cycle 2) were compared with baseline.

*Act*: A wider rollout and a proposed third PDSA cycle extending the proforma to acute medical and emergency department settings are discussed as future work. The stakeholders involved in the design and implementation included respiratory physicians, pleural clinical nurse specialists, and departmental clinical governance leads. Formal barriers to implementation (e.g., time constraints, competing documentation burden, and variable clinician engagement) were not systematically recorded, which represents a limitation of this evaluation.

Implementation and training

Prior to Cycle 2, clinicians and pleural nurses in the pleural service received a structured teaching session on the RED score and standardised pro forma, supplemented by a written quick-reference guide embedded in the clinical documentation template. No formal competency assessment was performed; adoption was monitored informally through case discussions at departmental pleural meetings during the implementation period. Pro forma completion rates and individual clinician adherence were not formally audited, which is acknowledged as a limitation in evaluating implementation fidelity.

Inclusion criteria

Cycle 1 (retrospective baseline): adults aged ≥18 years with a final diagnosis of malignant pleural effusion, confirmed by positive pleural fluid cytology or histopathology, or classified as malignant based on a high clinical probability in patients with negative cytology who were not candidates for thoracoscopy.

Cycle 2 (prospective intervention): adults aged ≥18 years with malignant pleural effusion confirmed following diagnostic work-up or classified as malignant based on high clinical probability in patients not candidates for thoracoscopy, provided they had not already been included in Cycle 1.

Exclusion criteria (both cycles)

Patients with a pre-existing indwelling pleural catheter prior to the index procedure, pleural infection/empyema, transudative effusion, or recurrent benign pleural effusion without evidence or clinical suspicion of malignancy were excluded.

Definitions of key outcome terms

Pre-test Probability

The clinician's overall pre-procedural estimate (high, intermediate, or low) that a pleural effusion was malignant, based on composite clinical, radiological, and historical features (e.g., known malignancy, weight loss, pleural nodularity, or thickening on imaging), rather than a validated numerical score.

Emergency Readmission/Unplanned Review

An unscheduled hospital admission or pleural clinic attendance occurring outside the pre-planned follow-up interval, directly attributable to symptoms or complications of known or suspected malignant pleural effusion.

Earlier-than-Planned Pleural Clinic Review

A pleural clinic attendance brought forward from its originally scheduled date because of clinical deterioration or new/worsening symptoms related to pleural effusion.

Definitive pleural management included successful indwelling pleural catheter insertion, chemical or surgical pleurodesis, or medical thoracoscopy, resulting in the resolution or planned long-term management of the effusion.

Ethics

This project was registered and approved as a quality improvement initiative by the institution's Clinical Governance Department. A formal Research Ethics Committee review was explicitly assessed and confirmed as not required in accordance with the UK Health Research Authority decision tool criteria for quality improvement/service evaluation projects using routinely collected clinical data, as this project did not involve a research intervention, randomisation, or allocation of care outside standard practice.

Data collection

Data for both cycles were extracted from electronic patient records by members of the clinical study team directly involved in patient care (Cycle 1: retrospective case note review; Cycle 2: prospective data entry at the time of clinical assessment). The data were not independently verified by a second reviewer. Missing or incomplete data fields were recorded as "unknown" or "not applicable" rather than imputed and are reported explicitly in the Results. Outcome assessors were not blinded to cycle allocation, as blinding was not feasible given the sequential before-and-after design; this was acknowledged as a potential source of ascertainment bias. Deaths and cases lost to follow-up before the three-month outcome assessment were identified from clinical records and reported separately.

Process and balancing measures

This evaluation did not formally capture process measures, such as pro forma completion rate or documented clinician adherence beyond informal case discussion; this is acknowledged as a limitation. Potential balancing measures, including unnecessary indwelling pleural catheter (IPC) insertion, procedural complications, pleural infection, length of hospital admission, and patient-reported dissatisfaction, were not systematically assessed, and we cannot exclude the possibility that a reduced aspiration frequency was offset by an increase in other forms of intervention-related morbidity.

Statistical analysis

Categorical variables were compared between cycles using Fisher's exact test, given the small sample sizes and sparse cell counts. The results are presented with risk differences, 95% confidence intervals, and odds ratios, where calculable. A two-sided P value <0.05 was considered statistically significant. Analyses were performed using Python 3.12.13 (SciPy library version 1.17.0) (Python Software Foundation, Wilmington, DE). No a priori power calculation was performed; this project was not designed or sized to detect statistically significant differences with confidence, and the reported P-values should be interpreted as exploratory rather than confirmatory.

## Results

Patient flow

A Consolidated Standards of Reporting Trials (CONSORT)-style patient flow diagram is presented in Figure 1, with a numerical summary in Table 1. In Cycle 1, 48 patients with confirmed malignant pleural effusion were included in the retrospective baseline cohort; 47 had complete three-month outcome data and one died before pleural effusion recurrence could be assessed, but all 48 were retained in the final analysis. In Cycle 2, 26 patients were included in the prospective intervention cohort; 21 had complete three-month outcome data, five had incomplete outcome data at the time of data lock, and five deaths during follow-up due to cancer progression were identified from clinical records, with all 26 retained in the final analysis using available data and outcome-specific evaluable denominators where necessary.

**Figure 1:**
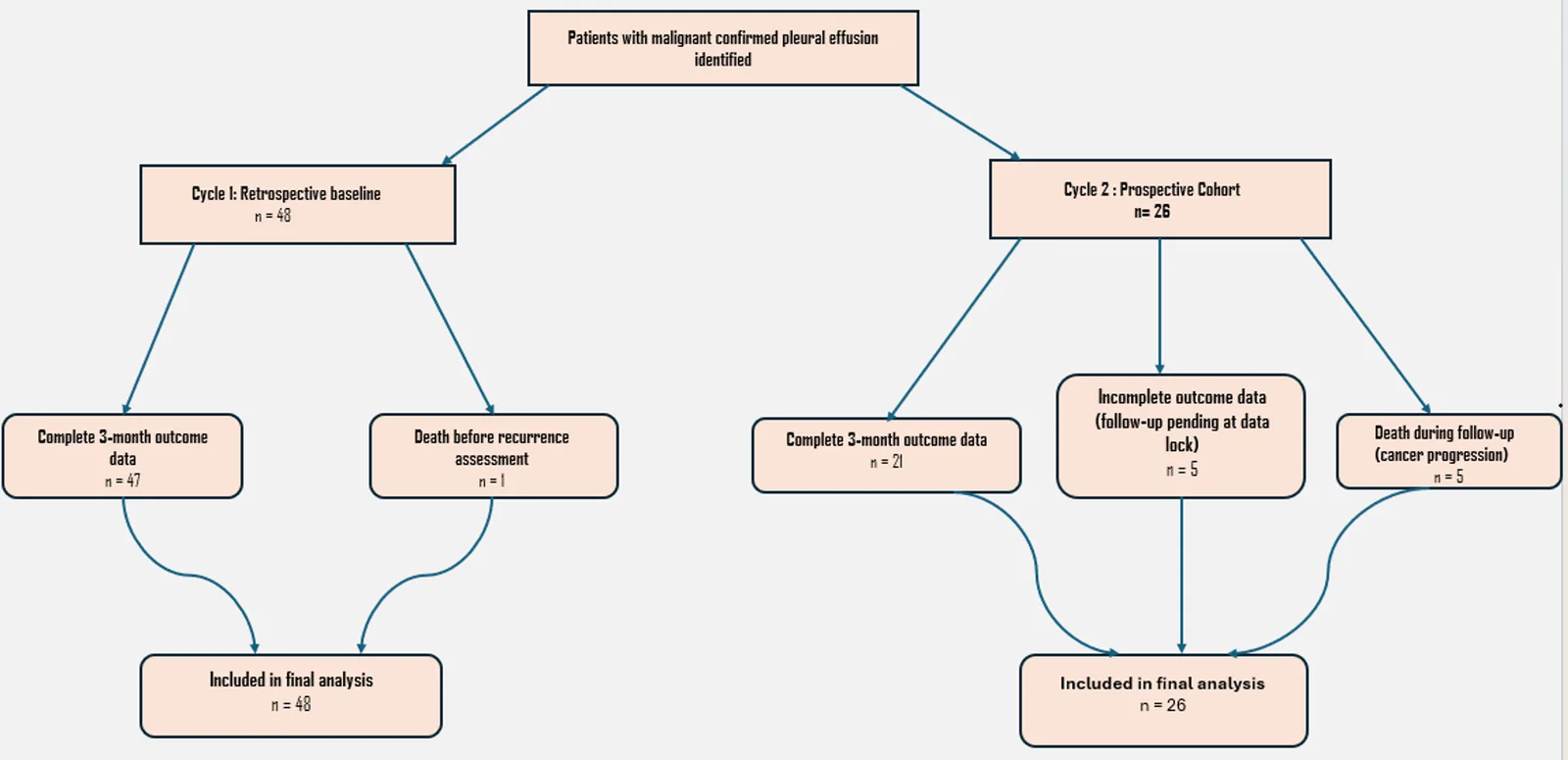
CONSORT-style patient flow diagram for the pleural quality improvement project Flow of patients with confirmed malignant pleural effusion through the retrospective baseline cohort (Cycle 1, n=48) and the prospective intervention cohort (Cycle 2, n=26). In Cycle 1, 47 patients had complete three-month outcome data and one died before recurrence assessment; all 48 were included in the final analysis. In Cycle 2, 21 patients had complete three-month outcome data, five had incomplete outcome data at the time of data lock, and five deaths during follow-up from cancer progression were recorded; all 26 were included in the final analysis. Abbreviations: IPC, indwelling pleural catheter; MDT, multidisciplinary team; CONSORT: Consolidated Standards of Reporting Trials

**Table 1:** Patient flow summary (CONSORT-style, adapted for a before-and-after QIP) Values presented as n or n/N (%). Screening/exclusion counts prior to final inclusion were not systematically logged in either cycle; this limits full transparency of case ascertainment. One Cycle 1 patient died before recurrence assessment. Abbreviations: CONSORT: Consolidated Standards of Reporting Trials; QIP: quality improvement program.

Stage	Cycle 1 (retrospective)	Cycle 2 (prospective)
Patients included	48	26
Patients with complete three-month outcome data	48 (100%)	22/26 (84.6%)
Patients with incomplete records at three months	0	4/26 (15.4%)
Deaths before planned intervention (among those with definitive intervention indicated)	Not systematically recorded	1/14 (7.1%)
Patients formally screened but excluded prior to inclusion	Not systematically recorded	Not systematically recorded

Unlike a randomised trial, this quality improvement project (QIP) did not involve a prospectively logged screening and exclusion pathway before cohort inclusion. Patients were identified retrospectively or prospectively through routine pleural service records according to the study case definition, and formal screening counts before final inclusion were not systematically recorded in either cycle. Figure 1 and Table 1 therefore provide transparency regarding cohort composition, completeness of follow-up, and deaths before interpretation of baseline characteristics and outcome comparisons.

Baseline characteristics

Table 2 compares the baseline characteristics between cycles, with statistical comparisons performed using Fisher's exact test. Performance status differed significantly between cycles, with a substantially higher proportion of Cycle 2 patients having ECOG scores ≥2 (87.5% vs. 41.7%, P<0.001). Age, sex, effusion size, and inpatient/outpatient settings did not differ significantly. The malignancy-type distribution and cytology positivity also differed descriptively between cohorts, and no data on disease stage, comorbidities, or systemic anticancer treatment were systematically collected, precluding formal adjustment for these potential confounders.

**Table 2:** Comparison of baseline patient characteristics between Cycle 1 and Cycle 2 Values presented as n (%) or n/N (%) using evaluable denominator where data were incomplete. P values calculated using Fisher's exact test where applicable. Cycle 2 patients had significantly worse performance status; malignancy type and cytology profile also differed descriptively, representing potential unadjusted confounders.

Characteristic	Cycle 1 (n=48)	Cycle 2 (n=26)	P value
Age ≥70 years	27 (56.3%)	13 (50.0%)	0.63
Female sex	30 (62.5%)	16 (61.5%)	1
Performance status ≥2	20/48 (41.7%)	21/24 evaluable (87.5%)	<0.001
Large effusion size	32 (66.7%)	15 (57.7%)	0.58
Inpatient setting at first procedure	20 (41.7%)	9 (34.6%)	0.62
Cytology positive	24 (50.0%)	10 (38.5%)	—
Cytology negative	19 (39.6%)	6 (23.1%)	—
Cytology suspicious	4 (8.3%)	6 (23.1%)	—
Cytology unknown/not performed	1 (2.1%)	1 (3.8%)	—
Most common primary malignancy	Breast (n=7), mesothelioma (n=7), gynecological (n=6)	Lung (n=8), breast (n=4), haematological (n=3)	—
Disease stage / comorbidities / systemic treatment documented systematically	No	No	—

Ultrasound and RED score reporting

Detailed thoracic ultrasound findings (septations, pleural nodularity, and diaphragmatic abnormalities) were not systematically extracted or tabulated in either cycle, and effusion size was recorded only categorically (large vs. small-to-moderate). Although intended as a core component of the Cycle 2 proforma, RED score values were recorded systematically for only a minority of patients and appeared inconsistently within free-text clinical comments rather than as a structured, extractable data field; documented examples included "RED score 24 (High)" and "RED score 26 (High),” while others recorded only a qualitative descriptor ("low RED score") without a numerical value. Due to incomplete and inconsistent documentation, we were unable to report a median RED score, full score distribution, stratification into high- versus low-risk groups, recurrence rates by RED score category, or any assessment of RED score calibration, discrimination, or comparison against clinician gestalt. This represents a substantial limitation in evaluating the specific contribution of the RED score to the observed outcomes, which is discussed further below.

Cycle 1: Retrospective Baseline (n=48)

Sixteen of the 48 patients (33.3%) experienced emergency admissions or earlier-than-planned pleural clinic reviews. Of these, 9/16 (56.3%) occurred within 14 days of the index procedure, 4/16 (25.0%) between 15 and 42 days, and 3/16 (18.8%) after 42 days postoperatively. Sixteen patients (33.3%) required more than two pleural procedures, whereas 32 (66.7%) required one or two pleural procedures. Definitive pleural management was achieved in 35 (72.9%) patients. Therapeutic pleural aspiration was the initial procedure in 40 patients (83.3%), and 28 patients (58.3%) were managed primarily through the elective pleural clinic (Table 3).

**Table 3:** Comparison of clinical outcomes between Cycles 1 and 2 Values presented as n (%) using evaluable denominators where specified. P values calculated using Fisher's exact test where applicable; risk differences and odds ratios reported for primary and secondary outcomes only, since these are the outcomes with sufficient event numbers for meaningful estimation. Given the small sample size, absence of randomization, uncontrolled before-and-after design, and lack of adjustment for confounding, these results should be interpreted as exploratory and hypothesis-generating rather than confirmatory or causal.

Variable	Cycle 1 (n=48)	Cycle 2 (evaluable denominator specified)	P value	Risk difference (95% CI)	Odds ratio
Emergency admission or unplanned pleural review	16 (33.3%)	2/26 (7.7%)	0.021	-25.6% (−42.5 to −8.8)	6.0
- Within 14 days	9/16 (56.3%)	1/2 (50.0%)	-	-	-
- Between 15 and 42 days	4/16 (25.0%)	1/2 (50.0%)	-	-	-
- After 42 days	3/16 (18.8%)	0/2 (0%)	-	-	-
Patients requiring >2 pleural interventions	16 (33.3%)	5/22 (22.7%)	0.415	−10.6% (−32.6 to +11.4)	1.7
Patients requiring 1-2 pleural interventions	32 (66.7%)	17/22 (77.3%)	-	-	-
Definitive pleural management achieved	35 (72.9%)	10/14 clinically indicated (71.4%)	-	-	-
Initial therapeutic pleural aspiration	40 (83.3%)	18 (69.2%)	-	-	-
Initial diagnostic pleural aspiration	-	7 (26.9%)	-	-	-
Initial chest drain	-	1 (3.8%)	-	-	-
Initial assessment in elective pleural clinic	28 (58.3%)	17 (65.4%)	-	-	-
Initial assessment during inpatient admission	20 (41.7%)	9 (34.6%)	-	-	-
Incomplete 3-month outcome records	0 (0%)	4 (15.4%)	-	-	-

Cycle 2: Prospective Intervention (n=26)

Following implementation, 2/26 patients (7.7%) experienced an emergency admission or earlier-than-planned pleural clinic review; one within 14 days and one between 15 and 42 days, with none beyond 42 days. Among the 22 patients with complete procedure-count data, 17 (77.3%) required one or two procedures, and five (22.7%) required more than two (Table 3).

Definitive pleural intervention was clinically indicated in 14/26 patients (53.8%); of these, 10 (71.4%) underwent definitive intervention, one died from malignancy progression before the planned intervention, one underwent diagnostic thoracoscopy pending MDT review, and one was technically unsuitable for IPC (Table 4).

**Table 4:** Definitive pleural intervention in the prospective cohort Percentages calculated using the 14 patients in whom definitive intervention was clinically indicated as the denominator. IPC: indwelling pleural catheter; MDT: multidisciplinary team.

Outcome	n	%
Patients assessed	26	-
Definitive intervention clinically indicated	14	53.8
- Underwent definitive intervention	10	71.4
- Did not undergo definitive intervention	4	28.6
- Died before planned intervention	1	-
- Diagnostic thoracoscopy pending MDT decision	1	-
- Technically unsuitable for IPC	1	-
- Incomplete record	1	-
Definitive intervention not indicated	12	46.2

## Discussion

This QIP demonstrated that implementing a structured pleural assessment pathway incorporating clinical pre-test probability, thoracic ultrasound findings, ECOG performance status, dyspnea assessment, and the RED score was associated with a marked reduction in clinically driven emergency admissions or earlier-than-planned pleural clinic review. Emergency presentations decreased from 33.3% in the baseline cohort to 7.7% following the implementation of the pathway. Although this study was not designed to establish causality, these findings suggest that a standardised approach to the initial assessment of patients with suspected malignant pleural effusion may improve the consistency of clinical decision-making and facilitate the early identification of patients requiring definitive pleural intervention.

The pathway also reduced the proportion of patients requiring repeated pleural procedures, with the percentage of patients undergoing more than two procedures falling from 33.3% to 22.7%. Importantly, this was not achieved by indiscriminately increasing definitive interventions. In the prospective cohort, definitive pleural intervention was clinically indicated in only 14 of 26 patients (53.8%), reflecting that many patients improved following therapeutic aspiration, had a low predicted recurrence risk, or were more appropriately managed conservatively because of advanced disease or end-of-life care needs. Among patients who required definitive intervention, 78.6% underwent definitive pleural management procedures. These findings highlight that the principal value of the pathway lies in improving patient selection rather than simply increasing the number of indwelling pleural catheters or pleurodesis performed.

The rationale for this approach is consistent with the current international guidelines. The British Thoracic Society Pleural Disease Guidelines recommend the systematic assessment of patients with malignant pleural effusion and early consideration of definitive pleural intervention for patients with recurrent symptomatic effusions. Similarly, the American Thoracic Society/Society of Thoracic Surgeons/Society of Thoracic Radiology (ATS/STS/STR) clinical practice guidelines advocate for individualised management based on symptoms, lung re-expansion, prognosis, and patient preference rather than repeated therapeutic aspiration alone [18,19].

Randomized controlled trials have consistently demonstrated the benefits of definitive pleural intervention in appropriately selected patients with MPE. The Second Therapeutic Intervention in Malignant Effusion Trial (TIME2) trial showed that indwelling pleural catheters provide symptom relief comparable to talc pleurodesis while reducing hospital stay [20]. The IPC-PLUS (indwelling pleural catheter; “PLUS” refers to the addition of talc sclerosant) study demonstrated that combining an indwelling pleural catheter with talc increased successful pleurodesis rates [21], whereas the Australasian Malignant Pleural Effusion-2 (AMPLE-2) trial showed that more aggressive drainage strategies improved pleurodesis outcomes and symptom control [22]. More recent reviews continue to support early risk-stratified management of malignant pleural effusion and emphasise individualised treatment strategies according to prognosis and expected recurrence of pleural effusion [23,24]. However, these studies evaluated different definitive treatment strategies rather than implementing a structured assessment pathway. Therefore, our project complements rather than duplicates the existing literature by focusing on the clinical decision-making process that precedes definitive intervention.

A particular strength of this project is the integration of several complementary clinical variables into a single, standardised assessment. Rather than relying solely on clinical judgement, the pathway combines pre-test probability, thoracic ultrasound findings, symptom burden, ECOG performance status, and the RED score to support individualised management planning. Standardising these assessments improved documentation, promoted consistent communication between clinicians and pleural nurses, and facilitated the earlier referral of patients considered most likely to benefit from definitive pleural intervention.

Importantly, this pathway is inexpensive and readily reproducible. No additional investigations were required beyond the standard clinical assessment and thoracic ultrasound. Implementation consisted primarily of introducing a structured pro forma supported by staff education and integration into routine pleural clinic practice. Consequently, this intervention could potentially be adopted by other pleural services with minimal additional resources.

These findings also provide a platform for further quality improvement. The proposed third PDSA cycle will extend the structured pathway beyond the pleural clinic into the Acute Medical Unit (AMU), same-day emergency care (SDEC), and Emergency Department (ED). Early assessment of patients presenting with known or suspected malignant pleural effusion would allow pre-test probability assessment and RED score calculation at the first pleural intervention, facilitating prompt referral to the pleural team and potentially reducing delays in definitive management before repeated hospital attendance occurs. Evaluating this broader pathway may demonstrate whether integrating acute medical services with specialist pleural care improves patient outcomes.

This study has several limitations. This was a single-center quality improvement project with a relatively small sample size and a non-randomised before-and-after design, limiting causal inference. Differences in patient characteristics between cycles, including performance status and underlying malignancy, may have influenced the outcomes, and multivariable adjustment was not possible. The intervention combined several components into a single structured pathway, preventing the assessment of the independent contribution of the RED score. In addition, patient-reported outcomes, procedural complications, balancing measures, and implementation metrics, such as clinician adherence, were not systematically collected. Finally, although the prospective cohort was recruited over a six-month implementation period, longer follow-up would be valuable to determine whether improvements are sustained and whether the pathway influences longer-term healthcare utilisation and patient-reported outcomes.

Overall, this QIP demonstrates that integrating clinical pre-test probability, thoracic ultrasound findings, and the RED score into a standardised pleural assessment pathway is feasible within routine clinical practice and is associated with fewer emergency presentations, fewer repeated pleural procedures, and more consistent selection of patients for definitive pleural intervention. Larger multicenter studies are required to validate these findings and determine whether standardised pleural pathways can improve outcomes across different healthcare settings.

## Conclusions

This single-centre, before-and-after QIP found a statistically significant reduction in unplanned emergency presentations and a non-significant numerical reduction in repeated pleural procedures following the introduction of a structured pre-test probability and RED score-guided proforma compared with a retrospective baseline cohort. However, given the small sample size, absence of randomisation and concurrent control, differing case mix between cohorts, incomplete RED score documentation, and lack of adjustment for confounding factors, these findings should be interpreted as preliminary and hypothesis-generating rather than as evidence that the intervention caused the observed improvements. We suggest, rather than conclude, that structured assessment pathways incorporating pre-test probability and prognostic scoring for MPE warrant further evaluation. Future work should include a larger, ideally multicentre and controlled or stepped-wedge evaluation, with pre-specified statistical analysis and power calculation, systematic capture of process and balancing measures, complete and structured RED score documentation, and inclusion of patient-reported outcomes before any recommendation for wider adoption of this specific pathway can be supported.
